# What names for covert awareness? A systematic review

**DOI:** 10.3389/fnhum.2022.971315

**Published:** 2022-08-05

**Authors:** Caroline Schnakers, Chase Bauer, Rita Formisano, Enrique Noé, Roberto Llorens, Nicolas Lejeune, Michele Farisco, Liliana Teixeira, Ann-Marie Morrissey, Sabrina De Marco, Vigneswaran Veeramuthu, Kseniya Ilina, Brian L. Edlow, Olivia Gosseries, Matteo Zandalasini, Francesco De Bellis, Aurore Thibaut, Anna Estraneo

**Affiliations:** ^1^Research Institute, Casa Colina Hospital and Centers for Healthcare, Pomona, CA, United States; ^2^College of Osteopathic Medicine, Western University of Health Sciences, Pomona, CA, United States; ^3^IRCCS Santa Lucia Foundation, Rome, Italy; ^4^Vithas Neuro Rehab Human Brain, Fundación Hospitales Vithas, Valencia, Spain; ^5^Neurorehabilitation and Brain Research Group, Instituto de Investigación e Innovación en Bioingeniería, Universitat Politècnica de València, Valencia, Spain; ^6^Centre Hospitalier Neurologique William Lennox, Ottignies-Louvain-la-Neuve, Belgium; ^7^GIGA-Consciousness, Coma Science Group, University of Liège, Liege, Belgium; ^8^Centre for Research Ethics and Bioethics, Uppsala University, Uppsala, Sweden; ^9^Science and Society Unit, Biogem, Biology and Molecular Genetics Research Institute, Ariano Irpino, Italy; ^10^Center for Innovative Care and Health Technology, School of Health Sciences, Polytechnic of Leiria, Leiria, Portugal; ^11^Ageing Research Centre, School of Allied Health, Health Research Institute, University of Limerick, Limerick, Ireland; ^12^Clínica Universitaria Reina Fabiola, Universidad Católica de Córdoba, Córdoba, Argentina; ^13^Subang Jaya Medical Center, Subang Jaya, Malaysia; ^14^Research Center of Neurology, Moscow, Russia; ^15^Faculty of Fundamental Medicine, Lomonosov Moscow State University, Moscow, Russia; ^16^Massachusetts General Hospital, Harvard Medical School, Boston, MA, United States; ^17^Centre du Cerveau, University Hospital of Liege, Liege, Belgium; ^18^Unità Spinale, Neuroriabilitazione e Medicina Riabilitativa Intensiva, Dipartimento di Medicina Riabilitativa, Azienda USL di Piacenza, Piacenza, Italy; ^19^IRCCS Fondazione Don Carlo Gnocchi, Florence, Italy; ^20^Neurology Unit, SM della Pietà General Hospital, Nola, Italy

**Keywords:** vegetative state, unresponsive wakefulness syndrome, minimally conscious state, covert awareness, cognitive motor dissociation, functional locked-in, non-behavioral MCS, consciousness

## Abstract

**Background:**

With the emergence of Brain Computer Interfaces (BCI), clinicians have been facing a new group of patients with severe acquired brain injury who are unable to show any behavioral sign of consciousness but respond to active neuroimaging or electrophysiological paradigms. However, even though well documented, there is still no consensus regarding the nomenclature for this clinical entity.

**Objectives:**

This systematic review aims to 1) identify the terms used to indicate the presence of this entity through the years, and 2) promote an informed discussion regarding the rationale for these names and the best candidates to name this fascinating disorder.

**Methods:**

The Disorders of Consciousness Special Interest Group (DoC SIG) of the International Brain Injury Association (IBIA) launched a search on Pubmed and Google scholar following PRISMA guidelines to collect peer-reviewed articles and reviews on human adults (>18 years) published in English between 2006 and 2021.

**Results:**

The search launched in January 2021 identified 4,089 potentially relevant titles. After screening, 1,126 abstracts were found relevant. Finally, 161 manuscripts were included in our analyses. Only 58% of the manuscripts used a specific name to discuss this clinical entity, among which 32% used several names interchangeably throughout the text. We found 25 different names given to this entity. The five following names were the ones the most frequently used: covert awareness, cognitive motor dissociation, functional locked-in, non-behavioral MCS (MCS^*^) and higher-order cortex motor dissociation.

**Conclusion:**

Since 2006, there has been no agreement regarding the taxonomy to use for unresponsive patients who are able to respond to active neuroimaging or electrophysiological paradigms. Developing a standard taxonomy is an important goal for future research studies and clinical translation. We recommend a Delphi study in order to build such a consensus.

## Introduction

After a severe brain injury, some patients do not fully recover consciousness and remain in a prolonged Disorder of Consciousness (DoC) such as the vegetative state/unresponsive wakefulness syndrome (VS/UWS) or the minimally conscious state (MCS). Patients in a VS/UWS open their eyes and present preserved autonomic functions, but they are not conscious and show only reflexive behaviors while the MCS is being characterized by the presence of inconsistent but clearly discernible behavioral signs of consciousness (e.g., visual tracking, command following) (The Multi-Society Task Force on Persistent Vegetative State, 1994; Giacino et al., 2002). More recently, the MCS has been subdivided in two clinical entities, MCS+ and MCS– (characterized by the presence/absence of command-following, intelligible verbalization, and intentional communication) supported by metabolic differences in areas associated with both consciousness and language (e.g., lower metabolism in the precuneus and thalamus and in the left middle temporal cortex in MCS-) (Thibaut et al., 2020). Prolonged DoCs are a relatively rare condition (estimated prevalence of 5,000–42,000 and 112,000–280,000 for VS/UWS and MCS, respectively, in the US) implying severe disability and complete dependence, which can last from 28 days to decades (Giacino et al., 2018).

Assessing behavioral signs of consciousness recovery in these patients can be challenging as it can lead to a misdiagnosis rate of approximately 40% when no standardized assessment tool is used (Schnakers, 2020). However, even with the most careful behavioral assessment, willful brain activity might still be missed in some of these patients. The emergence of Brain Computer Interfaces (BCI) which record and analyze brain signals to translate them into commands relayed to output devices that carry out desired actions without the intervention of neuromuscular output pathways has revolutionized our field and has led to the identification of a new clinical phenomenon (Annen et al., 2020). Indeed, for the past decade, clinicians have been facing a group of patients who are unable to show any behavioral sign of consciousness at the bedside but are able to respond to active neuroimaging or electrophysiological paradigms. The first report of such a phenomenon was published in 2006 and described the case of a young woman with severe brain injury diagnosed as being in a VS/UWS. When performing a mental imagery task (e.g., imaging playing tennis), her fMRI-related brain activity was similar to the one observed in healthy controls (Owen et al., 2006). Later, in a study using the same fMRI paradigm in a bigger sample (*n* = 54), two patients clinically diagnosed as being in a VS/UWS and three patients clinically diagnosed as being in a MCS were able to perform the task. One of these patients was able to correctly answer “yes” or “no” to autobiographical questions and therefore communicated by using either motor or spatial imagery (Monti et al., 2010). Since then, this phenomenon has been extensively reported in the literature. Two recent meta-analyses found that it can be observed in 14–17% of patients whose behavioral assessment suggested VS/UWS (Kondziella et al., 2016; Schnakers et al., 2020). The existence of this unique population has led the American Academy of Neurology and the European Academy of Neurology to introduce the importance of complementary techniques such as neuroimaging and electrophysiology when diagnosing patients with DoCs (Giacino et al., 2018; Kondziella et al., 2020).

However, even though researchers and clinicians recognize its existence, there is still no consensus regarding the nomenclature for this clinical entity (Owen, 2015). Finding a name for this phenomenon is nevertheless crucial to its clinical recognition and therefore to its diagnosis and to the development of a clinical management targeted to this population. Therefore, we performed a systematic review to 1) identify the terms used in the scientific literature to indicate the presence of this entity through the years, and 2) start an informed discussion regarding the rationale for these names and the best candidates to name this fascinating disorder.

## Materials and methods

### Inclusion and exclusion criteria

To be included in this systematic review, the following criteria were considered: (1) studies published in English between 2006 (year of the first publication on the topic) (Owen et al., 2006) and 2021, (2) peer-reviewed observational study (i.e., cross-sectional, longitudinal, retrospective or prospective) and reviews (reviews, systematic reviews, meta-analyses), (3) human subjects aged > 18 years old, (4) diagnosed as being in a VS/UWS or MCS (based on a standardized assessment scale such as the Coma Recovery Scale–Revised), and (5) evaluated using neuroimaging and/or electrophysiological active paradigms (defined as participants instructed to mentally perform a task). All etiologies and all clinical settings were included.

### Search methods

This systematic review was performed in accordance with the PRISMA (Preferred Reporting Items for Systematic Reviews and Meta-Analyses) guideline (Liberati et al., 2009). Search terms were generated in consultation with the Powell library at the University of California Los Angeles (Supplementary material). An electronic search of published studies was performed on both PubMed and Google Scholar in January 2021. The titles and abstracts of all articles in the search were divided among members (RF, EN, RL, NL, MF, LT, A-MM, SD, VV, KI, BE, OG, MZ, FB, AT, and AE) of the International Brain Injury Association (IBIA) Disorders of Consciousness Special Interest Group (DoC SIG) and then screened. Additional articles were manually searched by cross-referencing using the “cited by” function as well as by reviewing the reference section of the selected papers. Relevant articles from this initial screening were then gathered by CS. The same IBIA DoC SIG members were then asked to screen selected manuscripts and, if relevant, extract the following information: 1) are there specific name(s) used to designate the target clinical entity?, 2) if yes, which name(s)?, 3) is there any rationale for using such name mentioned in the text?

### Statistical analyses

Descriptive statistical analyses (i.e., measures of frequency such as count, percent) and qualitative analyses were used in this study using Microsoft Excel Spreadsheet Software.

## Results

**Figure 1:**
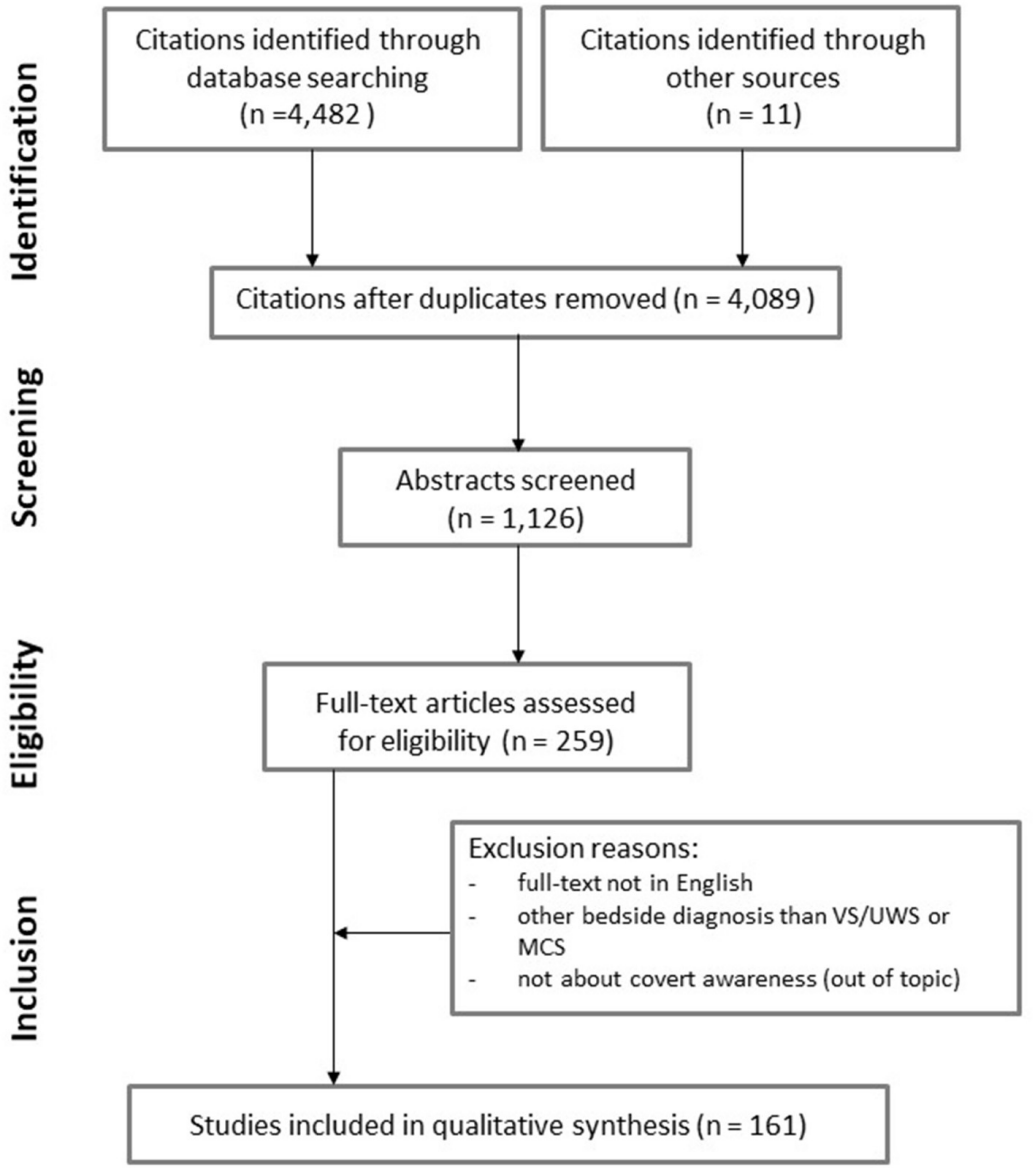
PRISMA flow diagram.

**Figure 2:**
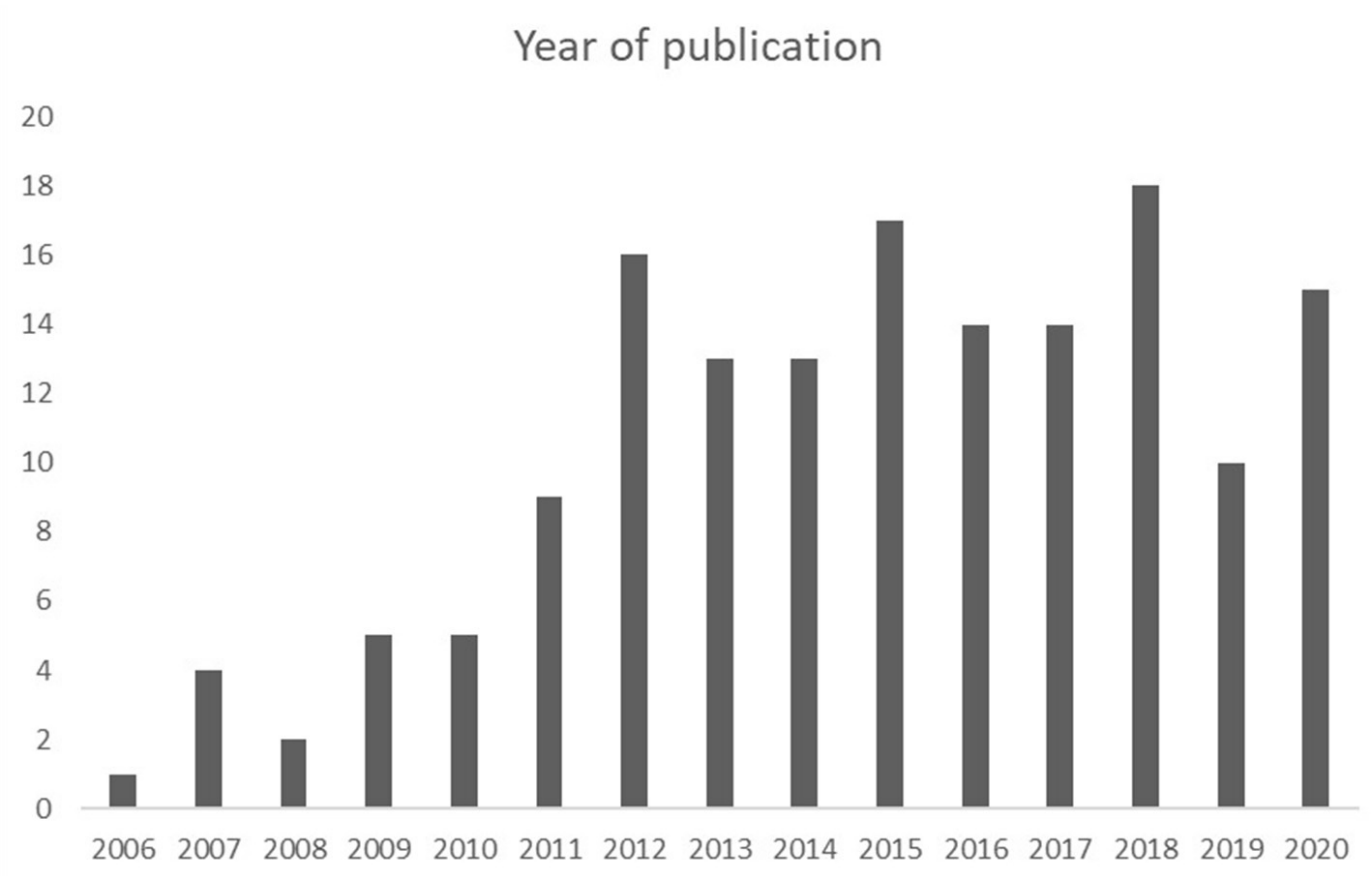
Year of publication (between 2006 and 2020) of the 161 relevant manuscripts included in our systematic review.

Our literature search on PubMed yielded 3,092 citations and on Google Scholar 1,390 citations (Figure 1). Eleven citations were manually added. After removing duplicates, a total of 4,089 citations were screened and led to 1,126 abstracts to be screened. A total of 259 manuscripts were screened, among which 161 were found to be relevant and included for data extraction (Figure 2 and Supplementary material).

Our findings show that 58% (*n* = 93) of the manuscripts used a specific name to designate the target clinical entity (see “Inclusion and exclusion criteria”), among which 32% (*n* = 30) used more than one name interchangeably across the manuscript. Only descriptive wording (such as “responders to active paradigm”) was used in the remaining 42% (*n* = 68). A list of 25 names was extracted, with 11 names used more than once. Some of these names had the same taxonomy root with “covert” being the most frequently used. The five following names were the most frequently used: covert awareness, cognitive motor dissociation, functional locked-in syndrome, non-behavioral MCS (MCS^*^), and higher-order cortex motor dissociation (Table 1). Publications of original authors using one of these 5 names were excluded to see how frequently each name was used by other authors (peers consensus). The results did show a slight change in terms of frequency of use in our final list of names with cognitive motor dissociation remaining the most used (Table 2).

**Table 1:** List of names given to our target clinical entity since 2006.

**Exhaustive list**	***n***
Akinetic mutism	1
Cognitive motor dissociation	26
Covert activity	1
Covert aspects of higher order function	1
Covert attention	1
Covert awareness	25
Covert behavior	1
Covert cognition	13
Covert cognitive abilities	1
Covert cognitive capacities	1
Covert cognitive process(ing)	3
Covert command following	4
Covert conscious awareness	3
Covert consciousness	19
Covert motor behavior	1
Covert residual cognitive function	2
Covert volitional brain activity	1
Covert volitional mental effort	1
Covert volitional neural activity	1
Functional disconnection syndrome	1
Functional locked-in syndrome	14
Functional minimally conscious	1
Higher-order cortex motor dissociation	4
LIS*	1
Non-behavioral MCS (MCS*)	8
**List by roots (cited>once)**	***n***
Covert*	**79**
***Covert awareness***	***25***
Covert conscious*	*22*
Covert cogn*	*18*
*Covert command following*	*4*
Covert volitional*	*3*
*Covert residual cognitive function*	*2*
**cognitive motor dissociation**	**26**
**functional locked-in syndrome**	**14**
non-behavioral MCS (MCS*)	**8**
**higher-order cortex motor dissociation**	**4**

The italic indicates subgroup of names. The bold indicates the names of the most frequently cited.

**Table 2:** Final list of names with provided definition and/or rationale.

**Final list of name**	**Author**	**Year**	**n**	**Used by other authors**
Covert awareness	Owen	2007	25 **(2)**	16 **(3)**
Functional locked-in syndrome	Giacino	2009	14 **(3)**	17 **(2)**
Non-behavioral MCS (MCS*)	Gosseries	2014	8 **(4)**	4 **(4)**
Cognitive motor dissociation	Schiff	2015	26 **(1)**	21 **(1)**
Higher-order cortex motor dissociation	Edlow	2017	4 **(5)**	3 **(5)**

The bold indicates the rating of names according to the number of citations.

## Discussion

The aim of our study was to identify the terms used to indicate the presence of covert awareness through the years, and to promote an informed discussion regarding the rationale for these names and the best candidates to name this novel clinical entity. Our results revealed that almost half of the manuscripts did not use any specific name, while a third used several names interchangeably. Such findings highlight the need to establish a consensus taxonomy for this clinical entity. As mentioned above, a final list of five candidates emerged. The rationale for each of them is discussed in this section, based on the selected manuscripts.

### Covert awareness and “Covert” related terms

The original article from Owen et al. (2006) described the phenomenon without providing a name (“…*some noncommunicative patients, including those diagnosed as vegetative, minimally conscious, or locked in, may be able to use their residual cognitive capabilities to communicate their thoughts to those around them by modulating their own neural activity*”). However, in an article published the year after, Owen and coworkers used, for the first time, the term “covert awareness” (Owen et al., 2007). The name was nevertheless only mentioned in the title and no explicit definition or explanation for using this term was provided in the text. Since then, other terms including “covert” (the most frequent being “covert consciousness” and “covert cognition”) have been used interchangeably to designate the presence of awareness, consciousness, top-down cognitive processing, volition, command-following or communication using task-based fMRI or EEG paradigms, in the absence of any behavioral signs of consciousness. An explicit rationale to use “covert awareness” or any of the “covert” related terms were not found in the articles published by the same authors and included in our search. The use of these terms seems therefore primarily descriptive.

### Functional locked-in syndrome

In 2009, Giacino and coworkers first introduced the term “functional LIS” observing that “… *commonly held notions about brain–behavior relationships should be revisited in this patient population…they clearly illustrate the wide discrepancy that may exist between observable behavior and the underlying neurophysiologic processes believed to support cognitive processing. Such findings also force us to consider the unsettling possibility that cognitive function may be at least partially preserved in this case, but lack a mode of expression as the consequence of severely dysfunctional sensory and motor systems. In a sense, these findings may reflect a “functional” LIS*…” (Giacino et al., 2009). In 2011, Bruno and coworkers more formally suggested the use of such term arguing that it emphasizes the dissociation between these patients' motor dysfunctions and their preserved higher cognitive functions as shown by functional imaging techniques (Bruno et al., 2011). In 2012, Laureys and Schiff specified that “*…this designation should be reserved, however, for patients who show consistent and reliable communication using non-speech and non-gestural communication through direct brain signaling”* (Laureys and Schiff, 2012).

The last publication emphasizes the importance of demonstrating communication in order to use such a term (and not only command-following and/or higher cognitive functions as Giacino and Bruno refers to). It nevertheless also complicates the use of such diagnosis since it requires showing consistent communication. This implies serial assessments using neuroimaging or electrophysiology, which, currently, might be practically complicated for clinicians. Moreover, considering the uncertainty about the full extent of residual cognitive function in such patients, using the term “LIS” which is characterized by relatively preserved cognition has been criticized (Owen, 2015; Schiff, 2015). Another criticism addresses the underlying neuropathology since LIS patients typically demonstrate specific lesions to the ventral pons which might not be the case in this population (Owen, 2015). Formisano and colleagues have been arguing in favor of using “functional” LIS as it underlines the distinction from “classical” or “complete” LIS and underlines the presence of a potential functional disconnection syndrome, as it is the case in diffuse axonal injury with associated supratentorial lesions (Formisano et al., 2013).

### Non-behavioral MCS

In 2014, Gosseries and coworkers criticized the use of “functional LIS” since the LIS is not a DoC, and since it could be confusing as well as a misnomer to use this term. Instead, the authors suggested that the use of non-behavioral MCS (or MCS^*^) is a more clinically accurate alternative as it is more descriptive and more consistent with other MCS terminology (i.e., MCS+ and MCS-) (Gosseries et al., 2014). In cases of dissociation between behavioral and neuroimaging testing, “*…especially in the case of patients who are diagnosed as being UWS by bedside testing but then diagnosed MCS with neuroimaging techniques*,” the use of MCS^*^ might therefore be a better description of the phenomenon (Gosseries et al., 2014). According to the authors, the term MCS^*^ can designate both VS/UWS patients who respond to active paradigms using neuroimaging and VS/UWS patients who demonstrate a brain activity at rest that is more compatible with MCS patients. One could wonder if distinct names should be given to each case scenario since they might reflect different levels of cognitive functioning and therefore different types of patients. Such a diagnosis (MCS^*^) would also only apply to VS/UWS that respond to active paradigms since the authors mention that “*…If patients in MCS- show command following during ancillary testing, they could be diagnosed as in MCS*+^*^.” Nevertheless, based on our search, such term (MCS+^*^) has not been used afterwards in the literature. Moreover, the term non-behavioral MCS has been criticized since it might not adequately describe the patients' residual cognitive abilities, because responses to complex active paradigms require preserved attention, language comprehension, and working memory which is currently assumed as being severely altered in MCS (Owen, 2015).

### Cognitive motor dissociation

Following the publication of Fernández-Espejo et al. (2015), who showed structural disconnection between thalamus and primary motor cortex (potentially leading to a deficit in motor expression) in one “covertly aware” patient (Fernández-Espejo et al., 2015), Schiff and coworkers wrote an editorial on these results and suggested the use of the term “Cognitive motor dissociation” (CMD) to account for “… *the sharp dissociation of a retained but unrecognized (covert) cognitive capacity in some severely brain-injured patients with non-purposeful or absent behavioral responses*” (as per the author, including patients in VS/UWS, MCS- or complete LIS who would demonstrate such response) (Schiff, 2015). This term constitutes a neutral description of the phenomenon and aims at being a general umbrella under which specific types of responses could be subcategorized (e.g., functional LIS, MCS^*^). However, some clarifications are needed regarding its definition since “*non-purposeful or absent behavioral responses*” can paradoxically not include MCS-. The inclusion of complete LIS might actually lead such a diagnosis to be applied to different types of pathologies and not to be specific to our population (such as amyotrophic lateral sclerosis or patients with severe Guillain Barrè syndrome which may present CMD in an advanced stage of the disease) (Zasler et al., 2019).

### Higher-order cortex motor dissociation

More recently, Edlow and coworkers also suggested the use of higher-order cortex motor dissociation (HMD), which they define as “… *functional MRI and EEG responses within association cortex (e.g., Wernicke's area) during passive language or music stimuli despite absence of behavioral evidence of language*” (Edlow et al., 2017). In this case scenario, patients who did not show any behavioral signs of receptive language but did show a relevant high-order cortical activation of the language network using a passive paradigm (i.e., passive exposure to a stimulus without instructing the patient to perform a task) might be in a HMD. The authors specified that this term should not be used for designating covert awareness (and so is distinct from previous terms such as CMD) but to label dissociation between neuroimaging and behavioral findings detected early on in the recovery process (e.g., in the intensive care unit).

Even though this systematic review leads us to consider a more confined list of names for diagnosing patients with covert awareness, it is clear that each term currently conveys confusion in terms of 1) the population it addresses (i.e., VS/UWS, MCS-, cLIS) and/or 2) the profile of responses it covers (i.e., command-following, communication and/or resting brain activity). Indeed, MCS^*^ seems to only include VS/UWS patients (Gosseries et al., 2014), while there is no clear mention of what bedside DoC diagnosis would functional LIS include (Giacino et al., 2009; Bruno et al., 2011; Laureys and Schiff, 2012). Additionally, functional LIS might designate patients either who only show “consistent communication” (based on Laureys and Schiff, 2012) or “higher cognitive functions” (based on Giacino et al., 2009; Bruno et al., 2011), while MCS^*^ might designate VS/UWS patients who respond to an active paradigm or who show resting state brain activity reflecting consciousness (Gosseries et al., 2014). A last dimension that might confuse clinicians is the method used to detect the phenomenon. Indeed, some authors mention only neuroimaging while others also include electrophysiology (Gosseries et al., 2014; Edlow et al., 2017). It is most likely that the method used would not lead to distinct terms but that should be clarified in the future. It should also be decided in the future whether covert awareness (as well as other similar terms such as covert consciousness and covert cognition) should have its own clear definition and should be used as a diagnosis. Beyond the need for clarity that was highlighted for each of these names, there is a need to decide whether one or several terms should be used for diagnosis or whether one umbrella term with associated subcategories should be used to fit specific patients' profile (e.g., higher cognition function vs. communications). Of course, this study has several limitations. Only two search engines were used for our systematic review (Pubmed and Google Scholar). Our systematic review was also limited to peer-reviewed publications and therefore excluded book chapters and dissertations. Only manuscripts in English were included which might have excluded names used in other languages. Finally, we focused our search on active paradigms and did therefore not include resting state paradigms or techniques such as Positron Emission Tomography (PET) or Transcranial Magnetic Stimulation (TMS or TMS-EEG), that might have been performed in that population. Future review of the literature could be more exhaustive and might lead to additional terms that were overlooked in this study.

## Conclusion

Since 2006, there has been no agreement regarding the taxonomy to use for patients who are able to respond to active neuroimaging or electrophysiological paradigms. Even though covert awareness was the first name to appear in the literature and has been one of the terms the most frequently cited, its use (and the use of other “covert” related names) has been primarily descriptive rather than diagnostic. “Functional LIS” was therefore the first name formally introduced to categorize our target clinical entity followed by “non-behavioral MCS” (MCS^*^) and “CMD.” In the future, expert consensus is needed to refine the definition of each of these names, but also to determine 1) if one or several names should be used to formally designate this phenomenon and, 2) in case of a taxonomy including several names (given the spectrum of patients' responses), if these names should reflect a continuum rather than clear separate categories. As a next step toward establishing consensus and creating a standardized taxonomy, we recommend a Delphi study which will be essential for optimizing future research studies and the clinical management of this phenomenon. Such study will have to consider the potential interpretation of a chosen taxonomy by the layperson and ensure that it does not convey confusing meaning and lead to unrealistic expectations for caregivers. The involvement of stakeholders in the choice of a specific taxonomy might help in decreasing such risk (Young and Edlow, 2021).

On the other hand, the clinical management of this population will certainly have to include serial state-of-the-art behavioral assessments as recommended by the American and European Academy Neurology to optimize the detection of consciousness at the bedside (Giacino et al., 2018; Kondziella et al., 2020). Tools such as flowcharts have recently been published to help clinicians implement recent guidelines and ensure high quality behavioral assessments before making a decision regarding the need of multi-modal assessments (Monti and Schnakers, 2022). Future studies should also address the risk of misinterpretation of multi-modal results (false positive) and develop protocols for clinicians to decrease this risk. Beyond the diagnosis, the integration of BCIs in the clinical management of covert awareness represents the future of this field, not only, in terms of establishing a communication with some of these patients, but also, potentially, in terms of facilitating cognitive rehabilitation in most of them (Annen et al., 2020). The clinical role of such technique should therefore be further investigated in the future in order to develop well-needed tools for clinicians who provide care to this challenging population.

## Data availability statement

The original contributions presented in the study are included in the article/Supplementary material, further inquiries can be directed to the corresponding author.

## Author contributions

CS and CB contributed to the study design, performed statistical analyses, drafted, and revised the manuscript. RF, EN, RL, NL, MF, LT, A-MM, SD, VV, KI, BE, OG, MZ, FD, AT, and AE participated in data collection, in interpreting data and in revising the manuscript. All authors contributed to the article and approved the submitted version.

## Funding

MF was supported by the European Union's Horizon 2020 Framework Programme for Research and Innovation under the Specific Grant Agreement (No. 945539) (Human Brain Project SGA3).

## Conflict of interest

The authors declare that the research was conducted in the absence of any commercial or financial relationships that could be construed as a potential conflict of interest.

## Publisher's note

All claims expressed in this article are solely those of the authors and do not necessarily represent those of their affiliated organizations, or those of the publisher, the editors and the reviewers. Any product that may be evaluated in this article, or claim that may be made by its manufacturer, is not guaranteed or endorsed by the publisher.
